# Policy in practice: Cystic Fibrosis Australia and Phage Australia surveys: understanding clinical needs and attitudes towards phage therapy in the cystic fibrosis community

**DOI:** 10.1093/sumbio/qvae036

**Published:** 2025-01-24

**Authors:** Stephanie Lynch, Holly Sinclair, Ameneh Khatami, Nicki Mileham, Jessica C Sacher, Jan Zheng, Ruby C Y Lin, Jonathan Iredell

**Affiliations:** Phage Australia at Westmead Institute for Medical Research, Westmead, NSW 2145, Australia; Faculty of Medicine and Health, The University of Sydney, Camperdown, NSW 2050, Australia; Westmead Hospital, Western Sydney Local Health District, Westmead, NSW 2145, Australia; Phage Australia at Westmead Institute for Medical Research, Westmead, NSW 2145, Australia; Faculty of Medicine and Health, The University of Sydney, Camperdown, NSW 2050, Australia; Westmead Hospital, Western Sydney Local Health District, Westmead, NSW 2145, Australia; Phage Australia at Westmead Institute for Medical Research, Westmead, NSW 2145, Australia; Faculty of Medicine and Health, The University of Sydney, Camperdown, NSW 2050, Australia; The Children’s Hospital Westmead, Sydney Childrens Hospital Network, Westmead, NSW 2145, Australia; Cystic Fibrosis Australia, North Ryde, NSW 2114, Australia; Phage Australia at Westmead Institute for Medical Research, Westmead, NSW 2145, Australia; Westmead Hospital, Western Sydney Local Health District, Westmead, NSW 2145, Australia; Phage Australia at Westmead Institute for Medical Research, Westmead, NSW 2145, Australia; Westmead Hospital, Western Sydney Local Health District, Westmead, NSW 2145, Australia; Phage Australia at Westmead Institute for Medical Research, Westmead, NSW 2145, Australia; Faculty of Medicine and Health, The University of Sydney, Camperdown, NSW 2050, Australia; Westmead Hospital, Western Sydney Local Health District, Westmead, NSW 2145, Australia; School of Medical Sciences, University of New South Wales, Sydney, NSW 2033; Phage Australia at Westmead Institute for Medical Research, Westmead, NSW 2145, Australia; Faculty of Medicine and Health, The University of Sydney, Camperdown, NSW 2050, Australia; Westmead Hospital, Western Sydney Local Health District, Westmead, NSW 2145, Australia

**Keywords:** cystic fibrosis (CF), phage therapy, antimicrobial resistance (AMR)

## Abstract

Cystic fibrosis (CF) is the most prevalent serious inherited disease in Australia, imposing significant health risks. CF is characterized by chronic lung inflammation and recurrent pulmonary infections that increase morbidity and premature mortality rates. The emergence of antimicrobial resistance (AMR) places further challenges on the treatment and management of CF, necessitating research into alternative strategies for treatment of bacterial infections. Bacteriophage therapy, involving bacterial-specific viruses, is a potential avenue for AMR infections in patients with CF. Existing literature supports the feasibility of phage therapy in CF but there has been a gap in investigating attitudes of the CF community including affected individuals and their caregivers, regarding phage therapy. Understanding perspectives and needs of the CF community is essential for successful implementation and acceptance of novel therapies including phage therapy. We conducted a survey that encompasses responses from 112 consumers from across Australia, comprising people living with CF (38.4%), parents of affected children (49.6%), carers (6.4%), and family members (3%). The findings showed a significant reliance on antibiotics with 51.4% requiring oral, 43.4% nebulized, and 11.4% intravenous antibiotics within the preceding 2 weeks. Respondents highlighted the availability of new treatments, duration of hospitalizations and costs associated with treatment as important priorities to address. Despite an awareness of phage therapy among 62.4% of respondents, 86.4% expressed interest in obtaining more information, primarily from medical staff (66.7%). Notably, 96.0% of respondents expressed willingness to participate in phage therapy trials. The results of this survey highlighted the CF community’s strong interest in advanced therapeutic approaches, specifically phage therapy. The findings reveal a notable recognition and acceptance of phage therapy as a viable treatment option for pulmonary infections associated with CF. This study addresses UN Sustainable Development Goal 3: Good Health and Wellbeing by advancing knowledge on alternative therapeutic strategies for managing AMR in CF. By exploring community attitudes towards bacteriophage therapy, the research promotes informed development and implementation of innovative, targeted treatments for CF-associated infections. These findings support the sustainable management of AMR, fostering better health outcomes and reducing reliance on traditional antibiotics, thereby contributing to long-term health resilience.

**Sustainability Statement**

This survey of cystic fibrosis (CF) patients aims to explore the perception of CF community towards phage therapy and implied potential of phage therapy as a sustainable microbiological approach to combat persistent lung infections. The results of this survey informs prescribers and phage product manufacturers to leverage naturally occurring bacteriophages to improve the health outcomes for individuals with CF but also addresses the growing concern of antibiotic resistance, which aligns with UN Sustainable Development Goal 3: Good Health and Well-Being. Phage therapy represents a promising alternative to traditional antibiotics, offering a targeted and environmentally friendly solution that minimizes ecological impact. Furthermore, our findings may contribute to UN Sustainable Development Goal 12: Responsible Consumption and Production by promoting innovative treatment strategies that reduce reliance on chemical antibiotics, thereby fostering sustainable healthcare practices.

## Introduction

Cystic fibrosis (CF) is one of the most prevalent inherited genetic disorders in Australia and it is estimated that the number of individuals living with CF will rise by 70.0% by 2025 in specific global regions (Elborn 2016, Dickinson and Collaco 2021). Data from the Australian CF Data Registry (ACFDR) currently records 3538 individuals living with CF, indicating an incidence of 1 in 2500 births (Lee et al. 2003, Ahern et al. 2021, Lin et al. 2023). CF is a multi-organ condition primarily characterized by pulmonary infections leading to progressive reduction in lung function (Elborn 2016, Dickinson and Collaco 2021). In children living with CF, predominant pathogens are *Haemophilus influenzae* and *Staphylococcus aureus*; however, as lung disease progresses, patients become colonized with *Pseudomonas aeruginosa* and other Gram-negative non-fermenting bacteria (Bhagirath et al. 2016). These pathogens frequently exhibit resistance to conventional antibiotics, necessitating prolonged courses of treatment that often do not achieve complete eradication (Perikleous et al. 2023). By adulthood, 80% of individuals become chronically colonized with *P. aeruginosa (*Parkins et al. 2018). A 2016 Australian study found that 31.0% and 35.0% of sputum isolates were multi-antibiotic resistant *P. aeruginosa* among paediatric and adult patients with CF, respectively (Smith et al. 2016). Notably, these resistant isolates showed high levels of resistance to frontline antibiotics, including aminoglycosides, β-lactams, and fluoroquinolones, which are commonly used in CF management. Currently there is no cure for CF, and treatments, including antibiotics, aim to manage symptoms, prevent complications and improve quality of life. However, rising antimicrobial resistance (AMR) places this vulnerable group at even greater risk of morbidity and mortality from infections that are resistant to conventional antibiotics. This highlights the critical need for research into development of alternative methods to treat bacterial infections in patients with CF.

Phage therapy or the therapeutic administration of bacterio(phages), has re-emerged as an antimicrobial strategy (Strathdee et al. 2023). Phages are bacterial viruses that recognize, attach to, and replicate within selective bacterial targets (Sulakvelidze et al. 2001). Due to this specificity to bacterial strains and inability to attack human cells, phages are generally regarded as safe. Phages can be used in cases where antibiotic options are limited or have associated toxicity (Ooi et al. 2019, Petrovic et al. 2020). Numerous compassionate cases have administered phage to treat a diverse range of bacterial pathogens in patients following lung transplantation or in those with CF (Kvachadze et al. 2011, Aslam et al. 2019, Dedrick et al. 2019, Lebeaux et al. 2021, Dedrick et al. 2022, Nick et al. 2022). These cases describe varying degrees of success in both microbiological and clinical outcomes, however, collectively support the safety and tolerability of phage therapy, without severe adverse events (Kvachadze et al. 2011, Aslam et al. 2019, Dedrick et al. 2019, Law et al. 2019, Lebeaux et al. 2021, Dedrick et al. 2022, Nick et al. 2022). Such reports highlight the potential of phage therapy as a viable approach to combat AMR bacterial infections in patients with CF. Despite these case reports (Kvachadze et al. 2011, Aslam et al. 2019, Dedrick et al. 2019, Law et al. 2019, Lebeaux et al. 2021, Dedrick et al. 2022, Nick et al. 2022) and the subsequent development of clinical trials for phage therapy in patients with CF (Tamma et al. 2022, Singh et al. 2023), clinical priorities of the CF community (patients and carers) and their attitudes towards phage therapy has yet to be explored.

To note, Australians have access to universal healthcare and patients with CF have access to subsidized and specialized disease management plans (https://www.cysticfibrosis.org.au/). This study aimed to survey both people living with CF and the broader CF community in Australia, including carers and family members of people with CF to understand the current burden of antimicrobial therapy in this population, and their perspectives regarding clinical trials involving phage therapy. It thus seeks to realign the priorities of phage therapy centres like Phage Australia by utilizing a co-designed framework to advance phage therapy specifically for CF. Ultimately, the survey results could be used to develop an advocacy framework for phage therapy to be presented to the Therapeutic Goods Administration of Australia.

## Methods

### Survey design and distribution

An anonymous online research survey was developed to understand the clinical needs of individuals and carers within the CF community in Australia, in particular, their attitudes towards clinical trials using new treatments, such as phage therapy. Ethical application was not sought, as specified by the WMA Declaration of Helsinki—ethical principles for medical research involving human participants. The risks and burdens were minimized due to the anonymous nature of this survey. Completion of the survey by the CFA community was considered implied consent. Participants were given the option to not have their responses included in the data analysis for this publication, however, no participants elected this option. The survey questions were designed in a close-ended format to facilitate statistical analysis using descriptive statistics. A team of clinicians, with expertise in caring for CF patients and experience in successfully treating patients with phage therapy, formulated the survey questions. In addition, these clinicians actively engage in phage therapy research. We also engaged Cystic Fibrosis Australia (CFA) in survey design, specifically framing questions to increase engagement. Thirteen single choice questions were input into Survey Monkey (https://www.surveymonkey.com). The survey could be completed in approximately 2 min.

The CF community was provided with a digital survey link through the CFA ‘Fight Fire with Fire’ newsletter (Supplementary S1). The newsletter also contained a brief introduction to phage therapy and to Phage Australia, to give context prior to completing the survey. The survey remained open for 4 months, aiming for at least 100 respondents (Supplementary S2).

The data was automatically collated on Survey Monkey, a password-protected platform accessible exclusively to study investigators. No identifiable information was collected. Participants had the opportunity to review responses prior to submission and modify before submission to ensure their comfort with participation and accuracy of data collected.

### Data analysis and visualization

Survey responses collated from Survey Monkey were downloaded into a password-protected CSV file. GraphPad Prism software was utilized where responses were visualized, including bar graphs depicting response percentages. The outcome of this survey (in the form of an accepted publication and a layman summary) will be communicated to the respondents through CFA communication channels, including e-mails and newsletter.

## Results

### Demographics of survey participants

Table 1 represents the demographic characteristics of the 125 participants. Geographically, there was a notable concentration of responses from individuals in NSW (New South Wales), VIC (Victoria), and WA (Western Australia), indicating a higher participation rate from these regions. The majority of respondents (98.4%) reported no Aboriginal or Torres Strait Islander ancestry. Of the 125 participants, 71.2% identified as female. Age distribution showed the majority (44.0%) falling within the 40–59 age group, followed by the 20–39 age groups (31.2%). This indicates a diverse representation across different age brackets within the surveyed population.

**Table 1.** Demographics of the 125 respondents of the survey.

	Number of responses (% of responses). Total responses *n* = 125
Respondent CF status	
Respondent has been diagnosed with CF	48 (38.40%)
Respondent is a parent of a child with CF	62 (49.60%)
Respondent is a family member of someone with CF (e.g. sibling, aunt/uncle)	4 (3.20%)
I am a carer of someone with CF	8 (6.4%)
Other	3 (2.40%)
Respondent age demographics	
13–19	18 (14.4%)
20–39	37 (29.6%)
40–59	54 (43.2%)
60–99	14 (11.2%)
Multiple selected	2 (1.6%)
Aboriginal and Torres Strait Islander origin	
None of the above	123 (98.40%)
Yes, Aboriginal	1 (0.80%)
Yes, Torres Strait Islander	1 (0.80%)
Yes, both Aboriginal and Torres Strait Islander	0 (0.00%)
Gender demographics	
Male	35 (28.00%)
Female	89 (71.20%)
Non-binary	1 (0.80%)
Geographical location	
NSW	31 (24.8%)
VIC	25 (20%)
WA	25 (20%)
QLD	13 10.4%)
SA	12 (9.6%)
ACT	11 (8.8%)
TAS	6 (4.8%)
NT	1 (0.8%)
No response	1 (0.8%)

### Antibiotic reliance and clinical priorities among CF patients

**Figure 1.**
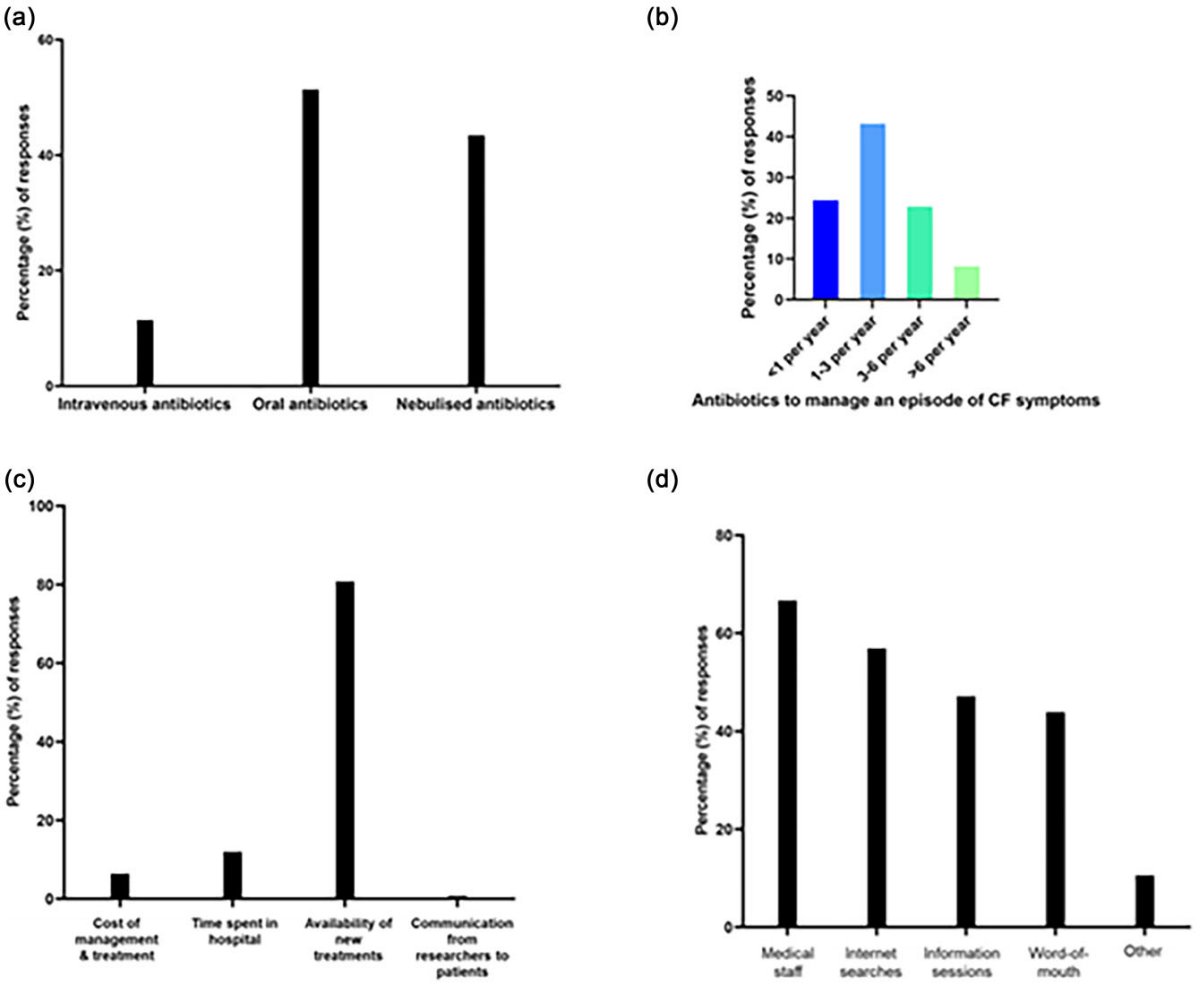
Overview of antibiotic use, treatment priorities, and information sources in Cystic Fibrosis Management. (a) Recent antibiotic use in the past 2 weeks for CF treatment. (b) Annual frequency of antibiotic courses for acute CF management. (c) Community-identified priorities for CF treatment and management. (d) Preferred sources of information on phage therapy from the CF community.

As shown in Fig. 1a, within the preceding two weeks, 51.4% required oral, 43.3% nebulized, and 11.4% intravenous (IV) antibiotics. Some respondents indicated concurrent use of oral, nebulized, or IV antibiotics within this 2-week period.

Almost half (43.9%) of respondents reported moderate antibiotic use (1–3 courses per year) to manage CF symptoms, while a quarter indicated infrequent use (<1 course per year) and a similar proportion indicating 3–6 courses per year were used (Fig. 1b). Responses also revealed that the majority (67.9%) of participants required less than one course of IV antibiotics to be given in hospital, annually.

The greatest priority among 80.80% of survey participants was placed on availability of new treatmen**ts** (Fig. 1c). Subsequently, time spent in hospital was a concern for 12.0% of participants. A smaller proportion of participants expressed concerns about the cost of therapies (6.4%) and communication from researcher to patients (0.80%).

### Awareness, engagement, and uptake of phage therapy within the CF community

The majority of respondents (62.4%) indicated an awareness of phage therapy, recognizing its potential as an alternative or adjunctive treatment to antibiotics for infections in CF.

Furthermore, the majority of survey participants (86.4%) indicated interest in learning more about phage therapy as an adjunct to antibiotics for CF treatment and/or management. Among those, the majority (66.7%) indicated they would rely on medical staff for more information (Fig. 1d).

An overwhelming majority (96.0%) of respondents expressed willingness to participate in a clinical trial investigating phage therapy. Similarly, 95.2% indicated an increased inclination towards engaging in phage therapy trials if government subsidized. Of the 125 respondents, only 5 answered ‘No’ when asked if they or the person they were caring for would consent to trial phage therapy. Notably, 4 of these 5 respondents were parents or caregivers.

## Discussion and conclusions

In response to limited knowledge about the CF community’s perception of emerging therapies, such as phage therapy, we conducted a survey to bridge this gap. Despite the longstanding use of phage therapy in medicine, and in treating infections in patients with CF, there has been limited exploration of this area. Our survey aimed to provide valuable insights into the CF community’s perspectives on this issue.

The demographics of the respondents of this survey align with recent summaries of the ACFDR, highlighting a median age of 20.2 years among individuals with CF, with children (<18 years) constituting 44.5% of the CF population (Ahern et al. 2021), consistent with the high proportion (almost 50%) of parent survey respondents. This highlights high engagement of parents among the CF community, and the importance of clinical trials to include paediatric populations. Geographic representation of survey responses was also largely consistent with ACFDR data on States of residence for individuals with CF (NSW = 1014 people, QLD = 908, and VIC = 724), although there was a lower-than-expected response rate from Queensland (Ahern et al. 2021).

Various pathogens, including *P. aeruginosa, H. influenzae, S. aureus*, and *Mycobacterium*, are associated with chronic infections in CF, leading to high antibiotic use in this population, either for prophylaxis or to manage exacerbations (Smyth and Walters 2001), and subsequent risk of developing multi-drug-resistant infections. Our survey responses confirmed this, with over half reporting oral antibiotic use within the preceding 2 weeks. Almost half of the participants reported requiring 1–3 courses of antibiotics per year, although the majority required less than one course (of presumably IV antibiotics) in hospital. These data are consistent with a previous study showing the majority of pulmonary exacerbations in patients with CF are managed with oral rather than IV antibiotics, particularly in young children (Wagener et al. 2013). Interestingly, a study investigating the impact of TRIKAFTA^®^ (Vertex Pharmaceuticals, Boston, MA, USA; elexacaftor / tezacaftor / ivacaftor) initiation on infection-related medical visits in patients with CF showed a significant and rapid decrease in antibiotic use following TRIKAFTA^®^ initiation (Miller et al. 2022). A limitation of our survey is that Trikafta use was not specifically enquired about, which could have provided valuable stratification of the data. Nebulized antibiotics were utilized by just under half of respondents consistent with other data indicating an increased use of long-term inhaled antibiotics in patients with CF (Moskowitz et al. 2008). Overall, the high burden of antibiotic use to manage exacerbations of CF symptoms was evident from survey responses, highlighting the significant impact chronic and recurrent chest infections have on the quality of life of patients with CF. Consequently, the survey results indicated a significant majority of respondents prioritized accessibility of new treatments for CF indicating an urgent need for alternative approaches to management of infections in patients with CF, particularly amidst the growing AMR crisis.

One advanced therapeutic approach already in use is phage therapy, the targeted use of phages against bacterial pathogens. Interestingly, over half of respondents had prior knowledge of phage therapy, in contrast to a 2023 study surveying the UK general public where only 13% were aware of it (Moskowitz et al. 2008, Wagener et al. 2013, McCammon et al. 2023). This suggests that people with CF or their families may be more informed about novel therapies. Additionally, our survey found there was a strong interest among respondents in learning more about phage therapy, with the majority selecting medical staff as their primary information source. However, it is important to note that medical professionals themselves may require comprehensive education on phage therapy including data from clinical trials, as 42.0% of Australian infectious disease physicians and 38.5% of Korean infectious disease physicians surveyed reported only moderate knowledge about phage therapy (Plymoth et al. 2023, Lee et al. 2024). A high level of willingness to participate in phage therapy trials was also expressed by survey respondents, with an even higher level of willingness to pursue new therapies if the government provided subsidies for treatment costs. This may reflect existing economic burdens associated with healthcare placed on this vulnerable population. To date, experience in using phage therapy for CF infections primarily comprises compassionate access cases (Kvachadze et al. 2011, Aslam et al. 2019, Dedrick et al. 2019, Law et al. 2019, Lebeaux et al. 2021, Dedrick et al. 2022, Nick et al. 2022). Thus, there is a critical need for increased access to clinical trials to systematically examine the effectiveness of phage therapy; however, only six clinical trials involving phage therapy and patients with CF are currently registered on clinicaltrials.gov (Singh et al. 2023).

The survey results provide valuable consumer insights for regulatory bodies in regard to antibiotic usage, and preferences for phage formulation and routes of administration. Data from clinical trials are needed to determine the most effective approaches, including the role of adjunctive antibiotics, duration of therapy, and the efficacy of single phage therapy versus a cocktail approach (Chan et al. 2013).

In addition, the survey results reflected that the key priorities of CF patients remain the same, i.e. management of respiratory health including adherence to prescribed medication, reduced reliance on antibiotics, and managing airway clearance and avoidance of respiratory infections. Utilizing phage therapy to improve quality of life that include shortened hospital stays and ameliorated daily functioning is implied.

This survey highlighted the importance of establishing a formalized knowledge-sharing network to educate and inform consumers, healthcare providers, scientists, and regulatory bodies about phage therapy. These measures can potentially accelerate the availability of phage therapy to individuals with CF.

## Review process

This paper was reviewed by an external peer reviewer via the journal’s standard process and was also reviewed by AMI’s policy editor David Oliver for its journal *Sustainable Microbiology*.

## Supplementary Material

qvae036_Supplemental_Files

## Data Availability

The data underlying this article are represented in the figures. For specifics, data will be shared on reasonable request to the corresponding author.
